# The Prevention of Post-Operative Gynecological Psychoses

**Published:** 1912-02

**Authors:** Herbert P. Cole

**Affiliations:** Mobile, Ala.; Lecturer in Gynecology, The Medical Department of the University of Alabama


### Journal-Record of Medicine

Successor to Atlanta Medical and Surgical Journal, Established 1855, and Southern Medical Record, Established 1870.

OWNED BY THE ATLANTA MEDICAL JOURNAL CO.

Published Monthly

*Official Organ Fulton County Medical Society, State Examining Board, Presbyterian Hospital, Atlanta, Birmingham and Atlantic Railroad Surgeons' Association, Chattahoochee Valley Medical and Surgical Association, Etc.*

EDGAR G. BALLENGER, M. D., Editor.
BERNARD WOLFF, M. D., Supervising Editor.
A. W. STIRLING, M. D., C. M., D. P. H., J. S. HURT, B. Ph., M. D.
GEO. M. NILES, M. D., W. J. LOVE, M. D., (Ala.); Associate Editors.
E. W. ALLEN, Business Manager.

COLLABORATORS

Dr. W. F. WESTMORLAND, General Surgery.
F. W. McRAE, M. D., Abdominal Surgery.
H. F. HARRIS, M. D., Pathology and Bacteriology.
E. B. BLOCK, M. D., Diseases of the Nervous System.
MICHAEL HOKE, M. D., Orthopedic Surgery.
CYRUS W. STRICKLER, M. D., Legal Medicine and Medical Legislation.
E. C. DAVIS, A. B., M. D., Obstetrics.
E. G. JONES, A. B., M. D., Gynecology.
R. T. DORSEY, Jr., B. S. M. D., Medicine.
L. M. GAINES, A. B., M. D., Internal Medicine.
GEO. C. MIZELL, M. D., Diseases of the Stomach and Intestines.
L. B. CLARKE, M. D., Pediatrics.
EDGAR PAULIN, M. D., Opsonic Medicine.
THEODORE TOEPEL, M. D., Mechano Therapy.
R. R. DALY, M. D., Medical Society.
A. W. STIRLING, M. D., etc., Diseases of the Eye, Ear, Nose and Throat.
BERNARD WOLFF, M. D., Diseases of the Skin.
E. G. BALLENGER, M. D., Diseases of the Genito-Urinary Organs.

VOL. LVIII. FEBRUARY 1912 No. 11

### Edition Chattahoochee Valley Medical and Surgical Association.

---

### THE PREVENTION OF POST-OPERATIVE GYNECOLOGICAL PSYCHOSES.

BY HERBERT P. COLE, M. D., MOBILE, ALA.

*Lecturer in Gynecology, The Medical Department of the University of Alabama.*

Post-operative nervous phenomena are not infrequent complications in gynecological cases. The severity of the psychoses has called forth an occasional commentary case report, though the subject in general has received little or no attention from the abler writers on gynecological problems.

The well-known tendency to derangement of the nervous mechanism in gynecological cases naturally predisposes to post-operative psychic sequelae, insomnia, hysteria, acute and chronic melancholia, neurasthenia and suicidal mania are a few of the unfortunate accidents that may arise following operation upon the female pelvis.

Permanency of cure in gynecological cases is frequently determined by the absence of post-operative psychic sequelae.

Frequently, of course, the nature of the pelvic pathology will obviate any possibility of operative relief of associated nervous lesions, and the nature of the gynecological disease may portend continued or exacerbated manifestation of the psychic disturbances.

We believe, however, that in a great majority of cases, the mild or even severe derangements may be prevented or in large part allayd by more careful attention to this feature in the management of gynecological cases.

There lies in the foreground of our minds, knowledge gained by experience, that we unconsciously apply to the management of our female cases with the object in view of making for the peace of mind of both the patient and operator, both through the period of hospital residence and through after life.

This preventive treatment naturally institutes itself with the first meeting or correspondence of patient and operator. Tactful management of correspondence, consultation, anemnesis and the examination, all conducted in a spirit of sympathy and assurance to the patient, frequently gains the confidence of the patient to such an extent that serious nervous outbreaks are obviated from the beginning.

A judicial selection of time and place for the operation. the association of a tactful nurse, a cool, quiet room, a short and comfortable pre-operative hospital residence are all factors in precluding a stormy convalescence.

The employment of a tactful reassuring anaesthetist in quiet surroundings carries the patient into the operation in proper mental condition for a short and peaceful post-operative experience. A short visit from the operator shortly after the patient regains consciousness; a short visit from one or two tactful relatives on the first day usually suffices to keep the patient in a normal mental condition.

Careful attention to the prevention or alleviation of post-operative pain, permitting free motion of limbs, latitude in change of posture, alcohol rubs or a most mild sedative will usually eliminate insomnia and thus the inception of a far more serious nervous sequelae.

Elimination of morphine therapy, the early and frequent use of the rectal tube, together with early evacuation of the bowels, removes tympanites as a serious etiological factor of psychic complications.

The substitution of a soft diet within a day or two after operation and a rapid return to the establishment of the patient's normal pre-operative diet is not only excellent physiologic therapy, but undoubtedly is a great factor in disassociating the patient's mind with the old ideas of invalidism usually conveyed by the liquid "foodless food" regime of former days.

The early institution of the back rest and removal to a chair within a few days after operation continue the dissipation of the "invalid idea." An early removal to the home not only establishes a shorter and more comfortable convalescence, but removes the patient from contaminating associations with other female patients whose "ida fixe" is a devilish desire to impregnate the minds of all newly operated cases with the hopelessness of their ultimate cure.

In conclusion, we feel the percentage of cases in whom we effect an ultimate and permanent cure depends largely upon personal study of the individual and constant endeavor to so conduct the case as to obviate the pathological psychic element in the convalescence.

202-204 Conti Street.

